# It’s about Time: Effects of Physical Exertion on Duration Estimates

**DOI:** 10.3390/jfmk4010006

**Published:** 2019-01-16

**Authors:** Britton W. Brewer, Lawrence O. Schwartz, Allen E. Cornelius, Judy L. Van Raalte, Edmundo L. Urbina, John S. Stubbs

**Affiliations:** 1 Springfield College, Department of Psychology, 263 Alden Street, Springfield, MA 01109 USA; 2 Ukiah Unified School District, 740 North Spring Street, Ukiah, CA 95482, USA; 3 Fielding Graduate University, 2020 De La Vina Street, Santa Barbara, CA 93105-3814, USA; 4 Wuhan Sports University, College of Health Sciences, No. 461 Luoyu Road, Wuhan 430079, China; 5 Massachusetts Youth Soccer, 512 Old Union Turnpike, Lancaster, MA 01523, USA; 6 Armed Forces Services Corporation, 2800 Shirlington Road # 350, Arlington, VA 22206, USA

**Keywords:** exercise, temporal judgments, time perception

## Abstract

Background: Task duration is a fundamental aspect of exercise, but little is known about how completed bouts of physical activity are perceived. Consequently, the purpose of the five experiments conducted for this investigation was to examine the effects of engaging in physical tasks on retrospective duration estimates with college student participants. Methods: Across the five experiments, participants were 113 college students (82 women, 31 men). In Experiments 1 and 2, participants provided duration estimates of a period spent engaging in physical activity or rest. In Experiments 3, 4, and 5, participants provided duration estimates of periods spent engaged in physical tasks of high intensity and low intensity. Results: In Experiments 1, 2, and 3, participants engaged in physical activity tended to perceive durations as shorter than participants at rest. When completing less familiar tasks (Experiments 4 and 5), however, participants recalled a high intensity bout of physical activity as lasting longer than a low intensity bout of physical activity of comparable duration. Cohen’s *d* values for physical activity effects on duration estimates ranged from 0.40 to 1.60. Conclusion: The findings, which partially support a contextual-change interpretation, suggest that factors, such as perceived exertion and task familiarity, affect retrospective duration estimates.

## 1. Introduction

Intensity and duration are fundamental descriptive elements of bouts of physical activity [1]. How intensity and duration of physical activity are perceived may have important implications for the extent to which people enjoy and adhere to physical activity regimens [2]. Whereas intensity of physical activity has been examined extensively from a psychological perspective in the form of perceived exertion [3], less empirical attention has been devoted to psychological aspects of physical activity duration. The perception of time, however, does have a rich tradition as the subject of psychological inquiry.

Research on time perception has been conducted for more than a century [4] using multiple methods under two main paradigms. As noted by Zakay and Block [5], commonly used methods include: (a) verbally estimating the duration of a time period (e.g., “How many seconds elapsed between the two tones of the bell?”); (b) producing a verbally requested time period by indicating when the period has elapsed (e.g., “Tell us when 30 s have elapsed.”); (c) reproducing a time period that has already elapsed (e.g., “Tell us when a time period as long as the tone you just heard has elapsed.”); and (d) comparing one or more reference time periods to a standard time period (e.g., “Tell us which of the three tones you just heard was exactly as long as the one you heard earlier.”).

The two main time perception paradigms involve giving duration estimates either prospectively or retrospectively. Under the prospective paradigm, research participants are made aware in advance of a given time period that they will be estimating the duration of that period. In contrast, under the retrospective paradigm, participants are not made aware that they will be estimating the duration of a time period until after the period has elapsed [5]. 

Results of studies across the diverse array of methodological approaches to time perception research can be compared through use of the duration judgment ratio, which is calculated by dividing the subjective (i.e., perceived) duration of a given time period by the objective (i.e., actual) duration of that period [6]. A duration judgment ratio greater than 1.00 corresponds to a time period being perceived as longer than it actually was (i.e., time “crawls”). A duration judgment ratio less than 1.00 corresponds to a time period being perceived as shorter than it actually was (i.e., time “flies”). A duration judgment ratio of 1.00 reflects a subjective estimate of time equal to the duration of the given time period.

The question of whether physical activity affects time perception has drawn periodic interest from investigators, all of whom used prospective methods. In the years since Weber [7] obtained findings indicating that duration judgment ratios tended to decrease as the amount of weight in an arm movement (kinesthesiometer) task, two distinct patterns of results have emerged. One group of studies has provided evidence bolstering the results of Weber [7], showing that increases in physical workload are associated with longer time productions (i.e., a decrease in duration judgment ratio—time “flies”) for a variety of tasks. Among the tasks for which such a pattern has been demonstrated include gripping a hand dynamometer [8,9], transferring pipes from one container to another [10], walking [11], and most recently, exercising on cycle and rowing ergometers [12]. These findings are consistent with the notion that physical activity occupies attentional resources that would otherwise be devoted to perception of temporal cues, resulting in perceptions of less time passing during a given interval than has actually passed [13].

A second group of studies has obtained results diametrically opposite those of the first group. Findings indicative of higher duration judgment ratios (i.e., time “crawls”) during physical activity than during rest have been obtained for cycling [14,15] and, when combined with a hot environment, treadmill running [16]. Thus, in contrast to the first group of studies, time was perceived as passing more slowly than it actually was when participants were engaging in physical activity than when they were at rest. The explanation given most consistently for this effect is that the physiological arousal induced by physical activity speeds up the pacemaker mechanism of people’s “internal clock” [17,18], which results in periods of time being perceived as longer that they actually are and productions of time being underestimates of actual duration. Other explanations include the possibility that the elevations in body temperature associated with physical activity influence time perception processes in the central nervous system and that the rapid motor movements involved in some forms of physical activity speed up the internal clock pacemaker mechanism biomechanically rather than by way of physiological arousal [15].

The effects of physical activity on time perception have not, to our knowledge, been examined under the retrospective paradigm. Retrospective estimates of physical activity duration are of potential importance because they contribute to the general impressions that individuals have of bouts of physical activity and may influence people’s subsequent attraction (or aversion) to the tasks in question [2]. As proposed by Block and Reed [19], retrospective time perception is influenced mainly by recollection of how many changes in cognitive context occurred during the interval, with more changes presumably reflecting longer duration. Consistent support for the contextual-change hypothesis has been obtained for retrospective duration estimates, with duration judgment ratios (i.e., perceived duration to actual duration) increasing as a function of the information-processing demands (i.e., cognitive load) experienced during the period to be recalled [6]. 

Engaging in physical activity induces physiological and behavioral changes in context relative to a resting state. Assuming these changes spill over into the cognitive realm and occupy attentional resources (as argued persuasively by Rejeski [20]), it would be expected from a contextual-change perspective [21,22] that retrospective duration estimates for a period of physical activity should be longer than those for a period of rest. To explore this possibility, we conducted an initial experiment with college student participants, the purpose of which was to examine the effect of physical activity on time perception under a retrospective paradigm. We hypothesized that the duration of a period of physical activity would be perceived as longer than that of a period of rest. Four follow-up experiments ensued, with each successive experiment involving a modification or extension of the previous study’s methods that was designed to elucidate or explain that study’s findings.

## 2. Materials and Methods

This investigation was conducted in accordance with the Declaration of Helsinki. The Springfield College Institutional Review Board gave initial approval to the procedures used in this research on 7 March 2007, under the title “Factors Affecting Exercise Task Perception.” Prior to their involvement in the research, all participants gave their informed consent for inclusion.

### 2.1. Experiment 1

#### 2.1.1. Participants

Participants were 17 undergraduate college students (14 women and 3 men) who ranged in age from 18 to 27 (*M* = 20.29, *SD* = 2.29) years. According to the reports of participants, the sample was predominantly White (90%).

#### 2.1.2. Instruments

A questionnaire item was used to assess how much time participants thought had elapsed during the sitting task.

#### 2.1.3. Procedure

The Institutional Review Board at the institution where the research was conducted approved the procedures used in this study. After participants completed an informed consent document, the experimenter measured the thigh length of participants, who were then randomly assigned to the WALL condition (*n* = 8) or the CHAIR (*n* = 9) condition by selecting a slip of paper with the name of the condition from inside a plastic bag. Participants in the WALL condition performed the wall sit (or “phantom chair”) task, in which they sat against a wall with their feet shoulder width apart and a thigh’s length from the wall so that their thighs were parallel to the floor. Participants in the CHAIR condition sat in a chair with their feet shoulder width apart and a thigh’s length from the back of the chair so that their thighs were parallel to the floor. After 25 s had elapsed, participants completed the questionnaire item asking them to indicate how much time they thought had elapsed during the sitting task, the beginning and end of which was denoted by the tone of a bell.

#### 2.1.4. Data Analysis

An independent samples t-test was performed to compare the duration estimates of the WALL condition with those of the CHAIR condition. An effect size was calculated in the form of a standardized mean difference (i.e., Cohen’s *d*) by dividing the difference between the WALL and CHAIR condition means by the pooled standard deviation. Duration judgment ratios were calculated for both conditions. 

### 2.2. Experiment 2

#### 2.2.1. Participants

Participants were 29 undergraduate college students (24 women and 5 men) who ranged in age from 18 to 26 (*M* = 20.07, *SD* = 2.36) years. According to the reports of participants, the sample was predominantly White (97%).

#### 2.2.2. Instruments

The questionnaire item used in Experiment 1 to assess how much time participants thought had elapsed during the sitting task was used again for the same purpose. Perceived exertion was assessed with Borg’s 6–20 RPE (rating of perceived exertion) scale, the psychometric soundness of which is well-documented [23].

#### 2.2.3. Procedure

The Institutional Review Board at the institution where the research was conducted approved the procedures used in this study. Procedures in Experiment 2 were identical to those in Experiment 1 with the exceptions that the duration of the sitting task was 40 s and participants also rated their levels of perceived exertion on Borg’s 6–20 RPE Scale upon completion of the task.

#### 2.2.4. Data Analysis

Two independent samples t-tests were performed to compare the duration estimates and RPEs of the WALL condition with those of the CHAIR condition. Cohen ‘s *d* was used as an indicator of effect size for the difference between the means for the WALL and CHAIR conditions. Duration judgment ratios were calculated for both conditions. A Pearson correlation coefficient was computed between duration estimates and RPEs (ratings of perceived exertion).

### 2.3. Experiment 3

#### 2.3.1. Participants

Participants were 29 undergraduate college students (16 women and 13 men) who ranged in age from 18 to 26 (*M* = 20.38, *SD* = 1.68) years. 

#### 2.3.2. Instruments

A brief questionnaire was used to obtain participants’ estimates of the duration of each cycle ergometer trial (i.e., the period of time between the end of the warmup and the beginning of the cooldown) and ratings of the levels of exertion, pain, discomfort, pleasure, and distraction that they experienced during the trial. Perceived exertion was assessed with Borg’s 6-20 RPE scale [23]. Single-item, 10-point (ranging from 0 to 9), Likert-type scales were used to assess perceptions of pain (anchors were no pain and pain as bad as it can be), discomfort (anchors were no discomfort and extreme discomfort), pleasure (anchors were not at all pleasurable and extremely pleasurable), and distraction (anchors were no distraction and extreme distraction).

#### 2.3.3. Procedure

The Institutional Review Board at the institution where the research was conducted approved the procedures used in this study. After completing informed consent and medical history documents, participants performed three trials on a Monark 864 cycle ergometer in which they did 2-min warmup at 70 revolutions per minute with 0.5 kg resistance followed by a 7.5 min trial of: (a) 0 revolutions per min with 0.5 kg resistance, (b) 70 revolutions per min with 1.0 kg resistance, or (c) 70 revolutions per min with 2.0 kg resistance. In pilot testing, the three trials corresponded to less than “very, very light” (i.e., no exertion) “fairly light” (i.e., light exertion), and “hard” (i.e., hard exertion) on Borg’s 6–20 RPE scale [23]. Each trial concluded with a 1-min cooldown at 70 revolutions per min with 0.5 kg resistance. The experimenter rang a bell at the end of the warmup and the beginning of the cooldown to denote the period for which duration estimates were sought. The order of the three trials was randomized and counterbalanced across participants by selecting a slip of paper listing the order in which participants would experience the conditions from inside a plastic bag. There was a 6-min interval between each trial. Immediately after each trial, participants filled out the brief questionnaire on which they estimated the duration of the trial (i.e., the period of time between the end of the warmup and the beginning of the cooldown) and rated the levels of exertion, pain, discomfort, pleasure, and distraction that they experienced during the trial.

#### 2.3.4. Data Analysis

Repeated-measures analyses of variance (ANOVAs) were performed on RPEs, duration estimates, and ratings of pain, discomfort, pleasure, and distraction. A Huynh-Feldt correction was used when Mauchly’s test of sphericity was significant. Planned contrasts were calculated between the no exertion condition and the other conditions and between the light exertion and hard exertion conditions. For each of the comparisons, Cohen’s *d* was calculated to indicate the effect size of the difference between condition means. Duration judgment ratios were calculated for all three conditions.

### 2.4. Experiment 4

#### 2.4.1. Participants

Participants were 12 undergraduate college students (9 women and 3 men) who ranged in age from 18 to 22 (*M* = 19.75, *SD* = 1.36) years. According to the reports of participants, the sample was predominantly White (91%).

#### 2.4.2. Instruments

A questionnaire item was used to assess which of the two hand crank trials participants thought was longer. Borg’s 6–20 RPE scale was used to assess perceived exertion [23]. Scales identical to those used in Experiment 3 were used to measure discomfort and pleasure, with the exception that 11-point scales (i.e., 0–10) were used instead of 10-point scales. Demographic information (e.g., age, sex, race) was obtained with a brief questionnaire. 

#### 2.4.3. Procedure

The Institutional Review Board at the institution where the research was conducted approved the procedures used in this study. After completing an informed consent document, participants completed an 83-second hand crank task under conditions of low exertion and high exertion. For the low exertion trial, participants were instructed to “Start turning the crank at a speed that is comfortable for you” and to “Please maintain a consistent rate throughout the trial” at the beginning of the trial and “Please keep turning the crank at a rate that is comfortable for you” 45 s into the trial. For the high exertion trial, participants were instructed to “Start turning the crank as rapidly as you can” and “Please maintain a consistent rate throughout the trial” at the beginning of the trial and “Please keep turning the crank as rapidly as you can” 45 s into the trial. A Megabrite self-powered flashlight was used for the hand crank task. A 60-second rest period separated the two trials. The order of the two trials was randomized and counterbalanced across participants by selecting a slip of paper listing the order in which participants would experience the conditions from inside a plastic bag. After the second trial, participants completed the brief questionnaire to indicate: (a) which trial they thought was longer; (b) rate the levels of exertion, discomfort, and pleasure that they experienced during the two trials; and (c) provide demographic information (e.g., age, sex, race). Borg’s 6–20 RPE scale was used to assess perceived exertion. Scales identical to those used in Experiment 3 were used to measure discomfort and pleasure, with the exception that 11-point scales (i.e., 0–10) were used instead of 10-point scales.

#### 2.4.4. Data Analysis

A series of paired samples t-tests was performed to compare participant’ ratings of exertion, discomfort, and pleasure for the low exertion and high exertion trials. For each of the comparisons, Cohen’s *d* was calculated to indicate the effect size of the difference between condition means. A sign test was performed to analyze participants’ responses to the item asking which of the two hand crank trials they thought lasted longer.

### 2.5. Experiment 5

#### 2.5.1. Participants

Participants were 26 undergraduate college students (19 women and 7 men) who ranged in age from 18 to 53 (*M* = 20.89, *SD* = 6.80) years. According to the reports of participants, the sample was predominantly White (88%).

#### 2.5.2. Instruments

The instruments were identical to those used in Experiment 4 with the exceptions that items asking for a specific duration estimate for each hand crank trial replaced the item asking participants to indicate which hand crank trial they thought was longer and the item pertaining to pleasure was eliminated.

#### 2.5.3. Procedure

The procedure was identical to that of Experiment 4 with the exceptions that participants, were given additional instructions to maintain a specific RPE of 6 to 8 (“Very, very light”) for the low exertion trial and 18 to 20 (“Very, very hard”) for the high exertion trial, gave specific duration estimates, and did not rate pleasure.

#### 2.5.4. Data Analysis

A series of repeated-measures analyses of covariance (ANCOVAs) with the condition presentation order as the covariate was performed on RPEs, duration estimates, and discomfort ratings for the low exertion and high exertion conditions. For each of the comparisons, Cohen’s *d* was calculated to indicate the effect size of the difference between condition means. Duration judgment ratios were calculated for both conditions. 

## 3. Results

### 3.1. Experiment 1

Exactly opposite to the hypothesized pattern of results, duration estimates were significantly shorter for the WALL condition (*M* = 21.88, *SD* = 7.53 s) than for the CHAIR condition (*M* = 36.11, *SD* = 9.93 s), ∆ = 14.23 s, *t* (13) = 3.29, *p* = 0.005, Cohen’s *d* = 1.60. Duration judgment ratios were 0.88 and 1.44 for the WALL and CHAIR conditions, respectively. Because we were unsure of the reason(s) for the unexpected finding, we conducted a second experiment, in which we extended the time of the wall sit task to increase the likelihood of creating contextual change and administered a measure of perceived exertion to quantify the workload during the wall sit task. Strong positive associations have been documented between perceived exertion and temporal variables during exercise [24].

### 3.2. Experiment 2

Means and standard deviations of all measured variables are presented in Table 1. The duration estimates of participants in the WALL condition did not differ significantly from those in the CHAIR condition, ∆ = 12.53 s, *t* (27) = 1.33, *p* = 0.20, Cohen’s *d* = 0.40. Duration judgment ratios were 1.05 and 1.37 for the WALL and CHAIR conditions, respectively. As one would expect, RPEs were significantly higher for the WALL condition than for the CHAIR condition, *t* (27) = 6.24, *p* < 0.001, Cohen’s *d* = 2.40. The mean RPE for the WALL condition fell midway between “fairly light” and “somewhat hard.” RPEs during the sitting task were inversely related to estimated duration (*r* = −0.37, *p* < 0.05). 

Taken together, the results of Experiments 1 and 2 suggest that over a brief period of time, physical activity in the form of the wall sit task produced retrospective duration estimates that were no longer—and possibly shorter—than a resting state. Although it is possible that the contextual change hypothesis does not apply to the effects of physical activity on retrospective duration estimates, it is also possible that the brevity and static nature of the wall sit task failed to produce the amount of contextual change required to elicit elongated duration estimates. Consequently, we conducted a third experiment, in which we used: (a) a longer, more dynamic physical task (cycle ergometry), with which multiple levels of perceived exertion could be implemented; and (b) to reduce variability in time perception across participants, a within-subjects design.

### 3.3. Experiment 3

Means and standard deviations of all measured variables are presented in Table 2. In a test of the physical exertion manipulation, the repeated-measures ANOVA with a Huynh-Feldt correction (due to a significant Mauchly’s test of sphericity) performed on RPEs showed a significant within-subjects effect across the three levels of physical exertion, *F* (2, 56) = 117.81, *p* < 0.001. Planned contrasts indicated significant differences in RPE between the no exertion condition and the light exertion and hard exertion conditions (*t* (56) = 13.74, p < 0.001, Cohen’s *d* = 3.67) and between the light exertion and hard exertion conditions (*t* (56) = 6.83, *p* < 0.001, Cohen’s *d* = 1.82). As intended, the means corresponded approximately to the verbal labels of “very, very light,” “fairly light,” and somewhere between “somewhat hard” and “hard” exertion, respectively, on Borg’s RPE scale.

In the analysis of primary interest, one observation was a distinct outlier (>4 standard deviations from the mean) and was deleted. The repeated-measures ANOVA with a Huynh-Feldt correction (due to a significant Mauchly’s test of sphericity) performed on duration estimates yielded a within-subjects effect across the three levels of physical exertion that was significant, *F* (2, 54) = 5.25, *p* = 0.02. Planned contrasts indicated a significant difference in duration estimates between the no exertion condition and the light exertion and hard exertion conditions (*t* (54) = 2.17, *p* = 0.03, Cohen’s *d* = 0.59), and between the light exertion and hard exertion conditions (*t* (54) = 2.40, *p* = 0.02, Cohen’s *d* = 0.65). Duration estimates during no exertion were significantly longer than those during light exertion and hard exertion, and light exertion duration estimates were significantly longer than hard exertion duration estimates on the cycle ergometer trial. The mean duration judgment ratios for the three levels of physical exertion were 1.15, 0.94, and 0.88, respectively, matching the trends found in Experiments 1 and 2 for duration judgment ratios to decrease with increases in physical exertion.

The repeated-measures ANOVAs with a Huynh-Feldt correction (due to a significant Mauchly’s test of sphericity) performed on pain scores, *F*(2, 56) = 75.17, *p* < 0.001, and pleasure scores, *F* (2, 56) = 7.77, *p* < 0.001, showed significant within-subjects effects across the three levels of physical exertion. Planned contrasts indicated significant differences in pain scores between the no exertion condition and the light exertion and hard exertion conditions (*t* (56) = 13.74, *p* < 0.001, Cohen’s *d* = 2.73) and between the light exertion and hard exertion conditions (*t* (56) = 6.83, *p* < 0.001, Cohen’s *d* = 1.81). Planned contrasts also indicated significant differences in pleasure scores between the no exertion condition and the light exertion and hard exertion conditions (*t* (56) = 2.93, *p* = 0.005, Cohen’s *d* = 0.78) and between the light and hard conditions (*t* (56) = 2.64, *p* = 0.01, Cohen’s *d* = 0.71). Mean pain scores increased and mean pleasure scores decreased steadily across the three levels of physical exertion. 

As with pain scores, the repeated-measures ANOVAs performed on discomfort scores, *F* (2, 56) = 52.67, *p* < 0.001, and distraction scores, *F* (2, 56) = 43.80, *p* < 0.001, showed significant within-subjects effects across the three levels of physical exertion. Planned contrasts indicated significant differences in discomfort scores between the no exertion condition and the light exertion and hard exertion conditions (*t* (56) = 8.78, *p* < 0.001, Cohen’s *d* = 2.35) and between the light exertion and hard exertion conditions (*t* (56) = 5.31, *p* < 0.001, Cohen’s *d* = 1.42). Planned contrasts also indicated significant differences in distraction scores between the no exertion condition and the light exertion and hard exertion conditions (*t* (56) = 8.65, *p* < 0.001, Cohen’s *d* = 2.31) and between the light exertion and hard exertion conditions (*t* (56) = 3.56, *p* < 0.001, Cohen’s *d* = 0.95). The mean discomfort score and mean distraction score increased steadily across the three levels of physical exertion.

Despite reporting that the trials became less pleasurable and more painful and discomforting, participants gave shorter retrospective duration estimates as the levels of exertion involved in the trials increased. Assuming that the elevations in perceived exertion, pain, discomfort, and distraction constitute changes in the cognitive context relative to the no exertion condition, the duration estimate findings in this experiment contradict the contextual-change hypothesis. It should be noted, however, that in obtaining duration estimates immediately after each trial on the cycle ergometer, the purely retrospective approach of Experiments 1 and 2 was lost. Although participants’ duration estimates for the first of the three trials were surely retrospective, it is likely that their duration estimates for the second and third were prospective. Consequently, we altered the design in Experiments 4 and 5 to ensure that temporal judgments for multiple levels of physical exertion were obtained retrospectively. Also, because familiarity with a physical task increases the accuracy of task duration estimates [25], an unfamiliar task was used in Experiments 4 and 5 to decrease the likelihood that participants could rely on previous task experience for temporal cues and to make the exertional properties of the task more salient.

### 3.4. Experiment 4

Means and standard deviations of all measured variables are presented in Table 3. In a test of the physical exertion manipulation, a paired-samples t-test performed on RPEs showed a significant within-subjects effect across the two levels of physical exertion, *t* (11) = 3.80, *p* = 0.002, Cohen’s *d* = 2.29. The mean RPEs for the low exertion condition and high exertion condition corresponded approximately to the verbal labels of “very light” and “somewhat hard” exertion, respectively, on Borg’s RPE scale.

In contrast to the findings of Experiments 1, 2, and 3, results indicated that 10 of the 12 participants rated the high exertion trial as lasting longer than the low exertion trial (sign test *p* = 0.02). Paired samples t-tests revealed that: (a) ratings of discomfort were significantly greater for the high exertion trial than for the low exertion trial, *t* (11) = 8.29, *p* < 0.001, Cohen’s *d* = 5.00; and (b) ratings of pleasure were significantly greater for the low exertion trial than for the high exertion trial, *t* (11) = 3.75, *p* = 0.003, Cohen’s *d* = 2.26. 

Experiment 5 was conducted to determine whether the apparent reversal of temporal judgments across levels of physical exertion observed in Experiment 4, in comparison with those documented in Experiments 1, 2, and 3, were replicable for duration estimates (rather than simply stating which trial lasted longer). It is possible that the unfamiliar task used in Experiment 4 may have been more suitable for generating exertion-related contextual change than the wall sit and cycle ergometer tasks used in Experiments 1, 2, and 3.

### 3.5. Experiment 5

Means and standard deviations of all measured variables are presented in Table 4. In a test of the physical exertion manipulation, the repeated-measures analysis of covariance (ANCOVA) performed on RPEs (controlling for condition presentation order) showed a significant within-subjects effect across the two levels of physical exertion, *F* (1, 24) = 14.52, *p* = 0.001, Cohen’s *d* = 4.67. The mean RPE for the low exertion condition was in the targeted range, whereas the mean RPE for the high exertion condition was slightly below the targeted range (but substantially higher than that for Experiment 4), and corresponded approximately to the verbal labels of “very light” and “somewhat hard” exertion, respectively, on Borg’s RPE scale.

Consistent with the results of Experiment 4, the repeated-measures ANCOVA (controlling for condition presentation order) revealed that duration estimates for the high exertion trial were significantly longer than those for the low exertion trial, *F* (1, 24) = 11.91, *p* = 0.002, Cohen’s *d* = 1.41. Duration judgment ratios were 1.23 and 1.14 for the high exertion and low exertion conditions, respectively. The repeated-measures ANCOVA (controlling for condition presentation order) also indicated ratings of discomfort were significantly greater for the high exertion trial than for the low exertion trial, *F* (1, 24) = 4.39, *p* < 0.05, Cohen’s *d* = 4.13.

## 4. Discussion

This investigation is the first of which we are aware to examine explicitly the effects of physical activity on duration estimates under the retrospective paradigm. The results of Experiments 4 and 5 support a contextual-change perspective [21,22] in that retrospective duration estimates for a period of vigorous physical activity on a novel task were perceived as longer than those for a period of less vigorous physical activity on the same task. From a theoretical standpoint, these results are presumably reflective of greater change in the cognitive and emotional context in the vigorous physical activity conditions in the two experiments. The results of Experiments 1, 2, and 3, however, are not compatible with—and, indeed, are in opposition to—a contextual-change approach. In those three experiments, the tendency was for periods of time spent engaging in physical activity to be perceived as shorter than those for comparable periods of time spent at rest. 

The inconsistencies in time perception documented in retrospective time perception as a function of physical activity in the current investigation mirror those documented in the literature pertaining to time perception in general [4,5] and to the effects of physical activity on prospective time perception in particular. Unfortunately, there are no obvious methodological distinctions between studies in which physical activity (or greater intensity physical activity) caused durations to be perceived as shorter than they actually were [7,8,9,10,11,12] versus those in which physical activity (or physical activity combined with a hot environment) caused durations to be perceived as longer than they actually were [14,15,16] to guide interpretation of the current findings.

Nevertheless, several tentative conclusions can be drawn about factors that potentially underlie the disparate findings across Experiments 1 through 5. Although task familiarity was not assessed directly, it is likely that the wall sit and cycling tasks used in Experiments 1, 2, and 3 were more familiar to participants than the hand crank task used in Experiments 4 and 5. Similarly, although the mean RPE given in Study 4 for the high exertion condition was comparable to that given in Study 3 for the hard exertion, the mean RPE for the high exertion condition in Experiment 5 was substantially higher than that given in any of the other experiments. Given that perceived exertion appeared to be associated with slowed or accelerated perceptions of time depending on the seeming novelty of the physical task, further research is needed to clarify the roles of exertion and task familiarity in shaping retrospective duration estimates. 

Also in need of clarification in future research is the role of emotional factors in retrospective duration estimates. The findings for discomfort, pain, and pleasure dovetailed with those for perceived exertion. Although the results of Experiments 4 and 5 (i.e., shorter duration estimates when pleasure was high and/or discomfort was low) are consistent with the old axiom “time flies when you are having fun,” the results of Experiment 3 (i.e., shorter duration estimates when pleasure was low and discomfort was high) directly contradict the axiom. The disparate findings for emotional variables in the current investigation speak to a more general need to operationalize more concretely and systematically the “cognitive context” in which retrospective temporal perceptions of bouts of physical activity are examined. Inquiry on the extent to which other psychosocial variables (e.g., physical activity enjoyment, self-efficacy) moderate or mediate the effects of physical activity on duration estimates could provide important information on that context.

A key consideration for future research pertains to the selection of physical tasks, task durations, and participant samples. Novel tasks may be needed to reduce the impact of prior task duration knowledge on physical activity duration estimates [25], but selection of such tasks may also diminish the external validity of the findings. Similarly, the task durations selected in the current investigation were considerably shorter than those of typical bouts of physical activity in the real world. Examining retrospective duration estimates of longer task durations in subsequent studies would enhance the external validity of the findings. With respect to the selection of participant samples, the college student samples used in the current set of experiments were adequate for an initial test of the effects of physical activity on retrospective duration estimates, but future investigations would benefit from the inclusion of samples consisting of individuals from populations of clinical interest (e.g., people who are sedentary). Sampling from clinically-relevant populations could facilitate exploration of the association between retrospective physical activity duration estimates and subsequent attraction and adherence to physical activity.

Another potential avenue for future research is to examine individual differences in time perception during physical activity and their correlates. The large standard deviations in Experiments 1, 2, 3, and 5 attest to the heterogeneity of duration estimates across participants. Even physically active individuals have difficulty estimating the amount of time they spend training in their preferred modes of exercise [26], and some “classic” (or robust) time perception effects outside of the realm of physical activity that have been well-established with aggregated group data do not necessarily hold up across individual participants [4]. Whether a given person tends to overestimate or underestimate time while engaged in physical activity may have important implications for that person’s attraction or aversion to physical activity [2]. 

In addition to the task and sample selection considerations identified above, there are two key limitations of the current investigation that should be addressed in future research. First, consistent with the literature on the effects of physical activity on time perception [13], all five experiments featured physical tasks in laboratory settings. Although laboratory tasks facilitate experimental control, the findings for such tasks may not generalize to physical tasks outside the laboratory. Examination of the effects of common forms of physical activity (e.g., walking, jogging) on duration estimates in free-living settings is warranted. Second, RPE was used as the sole means of assessing intensity of physical activity in the current investigation. Although the association of RPE with heart rate is robust [23,24], use of heart rate to assess intensity of physical activity in future research would provide an objective complement to the more subjective RPE. 

## 5. Conclusions

Overall, the findings of the current investigation partially supported the main hypothesis and echo the complex and sometimes-conflicting results of research on time perception across content domains [4,5], and highlight areas of ambiguity to be addressed in future studies of retrospective duration estimates specific to physical activity. The current investigation can serve as a point of departure for further examination of the ways in which physical activity affects temporal judgments and the factors associated with these perceptions, including the familiarity and exertional properties of the physical task. Exploration of the applied implications of differences in physical activity duration estimates warrants consideration.

## Figures and Tables

**Table 1:** Means and Standard Deviations of Estimated Duration and Perceived Exertion for the WALL and CHAIR Conditions in Experiment 2.

Variable	WALL		CHAIR	
	M	SD	M	SD
Estimated duration (sec)	42.14	21.99	54.67	28.18
Perceived exertion (RPE)	12.07	2.06	7.60	1.81

**Note:***n* = 29.

**Table 2:** Means and Standard Deviations of Estimated Duration, Perceived Exertion, Pain, Discomfort, Pleasure, and Distraction under Conditions of No Exertion, Light Exertion, and Hard Exertion in Experiment 3.

Variable	No Exertion		Light Exertion		Hard Exertion	
	M	SD	M	SD	M	SD
Estimated duration (min)	8.62	3.72	7.05	2.27	6.61	2.31
Perceived exertion (RPE)	7.52	1.64	11.21	1.68	14.17	2.98
Pain	0.55	1.21	2.45	1.86	4.79	2.16
Discomfort	0.93	1.98	2.83	1.97	4.86	2.34
Pleasure	4.86	3.00	4.28	2.00	3.00	1.98
Distraction	0.72	1.36	2.83	2.24	4.14	2.36

Note: *n* = 29 (*n* = 28 for Estimated duration (min)).

**Table 3:** Means and Standard Deviations of Perceived Exertion, Discomfort, and Pleasure under Conditions of Low Exertion and High Exertion in Experiment 4.

Variable	Low Exertion		High Exertion	
	M	SD	M	SD
Perceived exertion (RPE)	8.75	2.42	13.42	3.65
Discomfort	1.08	1.08	6.42	2.23
Pleasure	3.92	2.35	1.50	1.68

Note: *n* = 12.

**Table 4:** Means and Standard Deviations of Estimated Duration, Perceived Exertion, and Discomfort under Conditions of Low Exertion and High Exertion in Experiment 5.

Variable	Low Exertion		High Exertion	
	M	SD	M	SD
Estimated duration (sec)	94.62	55.66	102.31	45.26
Perceived exertion (RPE)	7.85	1.74	17.42	2.34
Discomfort	1.00	1.65	6.12	2.01

Note: *n* = 26.
